# Endoscopic Treatment of Gastric Band Prolapse

**DOI:** 10.1007/s11695-014-1253-7

**Published:** 2014-04-17

**Authors:** S. H. Kang, K. C. Kim, K. H. Kim

**Affiliations:** Department of Surgery, Seoul SKY Hospital, 36-1 Karakdong, Songpa-gu Seoul, 135-100 South Korea

**Keywords:** Endoscopy, Band prolapse, Reduction, Gastric band, Sutureless technique

## Abstract

Complications of laparoscopic adjustable gastric banding (LAGB) are well documented including migration, erosion, prolapse, infection, pouch dilatation, and gastric perforation. Band prolapse within the first 5 years after LAGB is observed in about 5 % of cases, requiring an operative procedure. Here we report our experience of endoscopic treatment of band prolapses. From December 2007 to December 2013, 1,347 consecutive patients (202 male, 1,145 female) underwent LAGB; 47 patients had band prolapses and 7 were treated by endoscopy. All patients were women (median age, 34 years). The mean preoperative body mass index was 38.3 ± 2.9 kg/m^2^. The mean duration to band prolapse after LAGB was 10.6 ± 5.6 months. The mean duration of endoscopy was 12 ± 3 min. One patient had recurrence of the prolapse 3 months after the first endoscopy and was treated by endoscopy again. There was no operative procedure required and no mortality. Endoscopic treatment of band prolapses is effective without the need for an operative procedure.

## Introduction

Laparoscopic adjustable gastric banding (LAGB) is a simple, safe, and effective procedure for treating morbid obesity. However, several complications after LAGB have been reported, such as band erosion, prolapse, gastric perforation, abscess, tube disconnection, port flip down, and infection. These complications could be the main cause of failure after LAGB. Revision weight loss surgery after failed LAGB might be considered occasionally [1].

Band prolapse is a significant and common late complication after LAGB [2]. Many surgeons advocate using gastrogastric sutures to prevent band prolapse [3]. However, band prolapse within the first 5 years even after using such a suturing technique is still observed in as many as 5 % of cases, requiring operative procedures [4]. Although the pars flaccida technique reduces the prolapse rate, the rate was still up to 5 % [5]. In addition, delayed diagnosis of a band prolapse can cause life-threatening complications such as pouch necrosis and gastric perforation [6].

We have performed a sutureless technique for the placement of Swedish adjustable gastric bands (Johnson & Johnson, New Brunswick, NK, USA) or MIDBAND™ bands (MID, Lyon, France). We treated some patients with a band prolapse using an endoscopic approach, and here we report our experience.

## Materials and Methods

### Patients

**Fig. 1:**
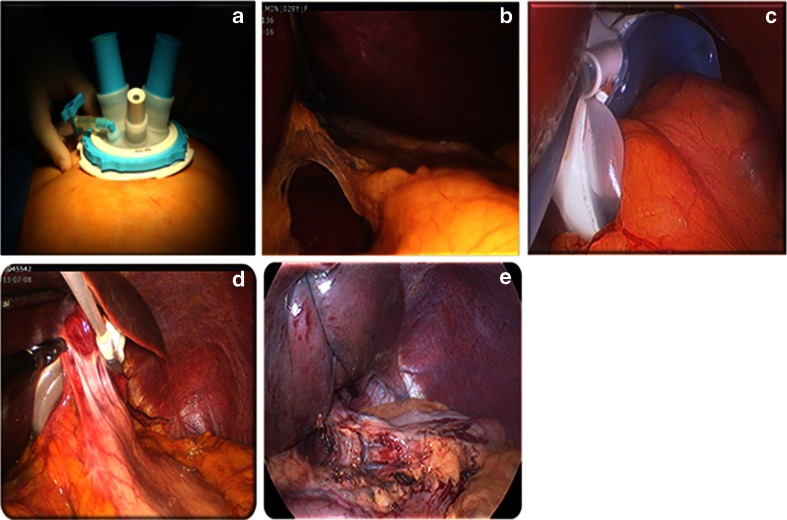
LAGB procedures. **a** We performed LAGB with an umbilical approach using Octoport™ (Dalim, Seoul, Korea). **b** The pars flaccida approach was done using a Goldfinger™ device (Johnson & Johnson, New Brunswick, NK, USA) as a liver retractor. **c** Even if the LAGB procedure was done without gastrogastric imbrication sutures, **d** fixation with fibrosis around the band could be observed 2 years after surgery. **e** Liver retraction was achieved using nylon sutures

From December 2007 to December 2013, a total of 1,347 consecutive patients (202 male, 1,145 female) underwent LAGB procedures using the pars flaccida technique with no imbrication suture and port placement under the anterior sheath of the rectus abdominis muscle (Fig. 1). From December 2007 to November 2011, 40 patients underwent revision surgery for a band prolapse. From December 2011 to December 2013, seven patients underwent endoscopic treatment of a band prolapse. We have followed them up for 2 years.

### Endoscopic Procedure

We evaluated the stomach by endoscopy before surgery, every 6 months after the LAGB procedure, and when the patients had symptoms of prolapse such as sudden abdominal pain and repeated vomiting.

**Fig. 2:**
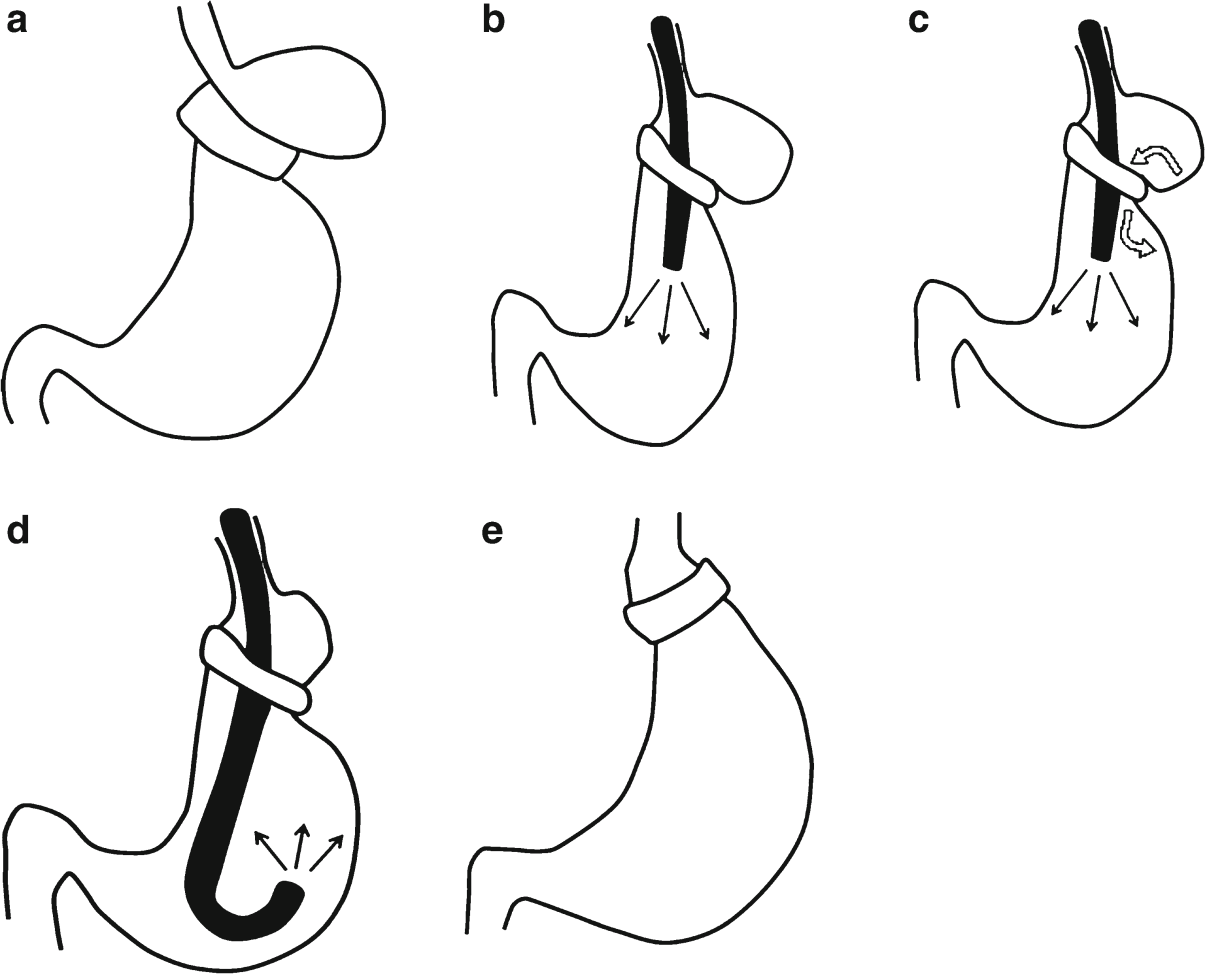
Illustrations for the endoscopic treatment of gastric band prolapse. **a** Prolapsed stomach. **b** If a prolapsed stomach is diagnosed, the endoscope is inserted into the stomach, which is then inflated with air (*black arrows*). **c** The prolapsed stomach is gradually reduced (*white curved arrows*) as the stomach is inflated with air. **d** After reduction is achieved, endoscopic examination of the entire lumen of the stomach can be performed. **e** Using this technique, the stomach could be restored to its normal position

**Fig. 3:**
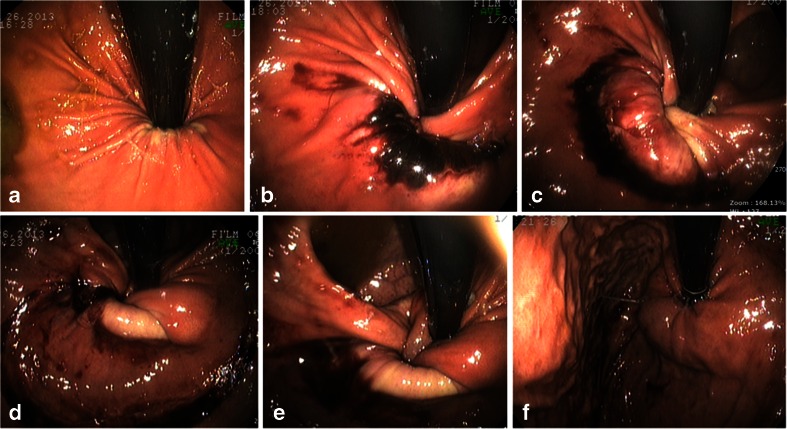
Endoscopic findings showed spontaneous reduction of the prolapse with air insufflation. **a** A prolapsed stomach is diagnosed using endoscopy. **b** After deflation of the band, the stomach is then inflated with air and is partially reduced. **c**, **d** The prolapsed stomach is gradually reduced. **e** The stomach is inflated with air then fully reduced so that the band is finally in its normal position. **f** After reduction, we examined the entire lumen of the stomach

The endoscopic procedure was performed after deflation of the band when the patient had these symptoms of prolapse. Band prolapse was diagnosed by X-rays after the patient has swallowed a barium contrast medium or by endoscopy. When diagnosed with band prolapse, the patient was sent to the endoscopy room under intravenous anesthesia. If we found a prolapsed stomach pouch, we inserted an endoscope and then inflated the stomach with air. The prolapsed stomach was gradually reduced as the stomach was inflated with air (Fig. 2). The stomach was fully reduced, and finally the band was returned to its normal position. After reduction, we examined the entire lumen of the stomach to check for normalcy (Fig. 3).

## Results

All seven patients were female. The patients’ ages ranged from 22 to 45 years (median, 34 years). The preoperative body mass index ranged from 35 to 43 kg/m^2^ (mean, 38.3 kg/m^2^). The duration to band prolapse after LAGB ranged from 1 to 18 months (mean, 10.6 months). The duration of endoscopy ranged from 9 to 15 min (mean, 12.3 min; Table 1).

**Table 1:** Clinical outcomes

Case	Gender/age (years)	BMI	Type of band	Period after LAGB (months)	Duration of endoscopy (min)
1	F/34	37	SAGB	13	14
2	F/22	39	SAGB	1	9
3	F/43	35	MID	7	11
4	F/37	41	SAGB	18	13
5	F/28	37	SAGB	15	12
6	F/45	43	SAGB	9	15
7	F/29	36	MID	11	12

*BMI* body mass index, *F* female, *MID* MIDBAND™, *SAGB* Swedish adjustable gastric band

The first adjustment of the band after reduction was done at 1 month. One patient had recurrence of the band prolapse 3 months after the first endoscopic treatment and was also treated by endoscopy (case 3, Table 1). We followed her up for 1 year without another recurrence of the band prolapse. There was no other operative procedure required and no mortality.

## Discussion

Since its first introduction in 1993, LAGB has been accepted as a reliable surgical option for treating obesity, thanks to its safety, minimal invasiveness, and effectiveness. However, bariatric surgeons have largely abandoned LAGB since 2008, and the number of procedures has declined because of adverse complications [7].

When a LAGB procedure is performed, the band should be placed around the upper part of the stomach, approximately 1 to 2 cm below the gastroesophageal junction to allow normal functions, limiting food intake and slowing the emptying of stomach contents into the duodenum. Surgical techniques have been suggested to lower the incidence of gastric prolapse, such as reduction of the size of the gastric pouch, the appropriate placement of gastrogastric sutures, and correct positioning of the posterior aspect of the band. The main advantage of gastrogastric suturing in LAGB is prevention of band prolapse [8, 9].

Band prolapse is a significant and common late complication after LAGB. The incidence of band prolapse within the first 5 years is still as much as 5 %, requiring operative procedures. It can occur anteriorly or posteriorly. The common symptoms and signs of band prolapse include food intolerance, vomiting, abdominal pain, reflux esophagitis, pouch dilatation, dehydration, and muscle weakness [10].

Band prolapse can be diagnosed by X-rays after the patient has swallowed a barium contrast medium or by endoscopy [11, 12]. A delay in diagnosis can cause hypokalemia with or without loss of consciousness, gastric perforation, or pouch necrosis leading to death [13]. As band prolapses do not respond to conservative measures, many surgeons advocate surgical options including repositioning of the band, replacement with a new one, or even removal of the band [14].

We have performed LAGB without imbrication sutures, and this makes the procedure much simpler and reduces the risk of complications during the initial or any potential revision surgery. Not all anterior band prolapses were reduced by band deflation and endoscopic approach. When we found any sign of infection or perforation inside the stomach, we fixed it operatively. Otherwise, almost all band prolapses after such sutureless LAGB without delay in diagnosis can be fixed easily with an endoscopic procedure. We could treat the patient without operative procedures even in case of a recurrent prolapse.

## Conclusion

Band prolapse is a significant and common late complication after LAGB. Endoscopic treatment of band prolapse is safe and feasible without requiring an operative procedure.
